# Comparison between a New Vaccine Virus and That of 1836

**Published:** 1845-04

**Authors:** 


*Comparison between a new Vaccine Virus and that of* 1836. M. Fiard has communicated to the Academy of Sciences, Paris, the result of experiments which he has made, in order to establish the differential characters of the development, march, and duration of the vaccine matter recently discovered by M. Magendie and himself, compared with that of 1836.

In one *arrondissement*, he inoculated 351 persons with it. It appears to be more active than the old, and to possess a greater facility of transmission in man. From these experiments, M. Fiard is of opinion that it is not, as has hitherto been thought, the greater or lesser development of the vaccine pustules on the eighth or ninth day, which essentially demonstrates the degeneration of the virus, but the regular and continued progress, and especially the duration of the eruption, which indicates the various degrees of this degeneration. It is more particularly, as in small-pox, the shortening of the regular period of the eruption, which denotes the attenuation of, or decrease of efficacy in, the virus collected from the cow, and artificially transferred and kept up in the human subject.

Tables containing the comparative facts, with regard to the development, march, and duration of the new vaccine lymph of 1844, and that of 1836, in the same child, show that, till the eighth day, there is no difference; but, on the ninth day, the desiccation of the old pustules commences, and is complete on the thirteenth or fourteenth day. The progress and development of the new, on the contrary, are slower, and desiccation is not complete till the sixteenth or seventeenth day, thus showing a difference of from three to four days.

Jenner's vaccine lymph, after thirty-nine years transmission from man to man, compared, in 1836, to the lymph of that period, dried upon the twelfth day; while that of 1836, like that of 1844, did not complete its desiccation till the seventeenth day. That of 1836, in the present day, after eight years sojourn in the human subject, compared to that of 1844, which does not dry up till the seventeenth day, desiccates on the thirteenth or fourteenth day, the eruptive duration thus losing three or four days. — *Med. News*, from *Prov. Med. and Surg. Jour.*, *Dec.* 18, from *Gaz. Méd. de Paris.*

